# Effect of eicosapentaenoic acid, protein and amino acids on protein synthesis and degradation in skeletal muscle of cachectic mice

**DOI:** 10.1038/sj.bjc.6601981

**Published:** 2004-06-22

**Authors:** H J Smith, N A Greenberg, M J Tisdale

**Affiliations:** 1 Pharmaceutical Sciences Research Institute, Aston University, Birmingham B4 7ET, UK; 2 Novartis Nutrition, 1541 Park Place Blvd., Minneapolis, MN 55416, USA

**Keywords:** protein synthesis, protein degradation, muscle, cachexia

## Abstract

Atrophy of skeletal muscle reduces both the quality and quantity of life of patients with cancer cachexia. Loss of muscle mass is thought to arise from a reduction in protein synthesis combined with an enhanced rate of protein degradation, and few treatments are available to counteract this process. Eicosapentaenoic acid (EPA) has been shown to attenuate the enhanced protein degradation, but to have no effect on protein synthesis. This study examines the effect of EPA combined with a protein and amino-acid supplementation on protein synthesis and degradation in gastrocnemius muscle of mice bearing the cachexia-inducing MAC16 tumour. Muscles from cachectic mice showed an 80% reduction in protein synthesis and about a 50-fold increase in protein degradation compared with muscles from nontumour-bearing mice of the same age and weight. Treatment with EPA (1 g kg^−1^) daily reduced protein degradation by 88%, but had no effect on protein synthesis. Combination of EPA with casein (5.35 g kg^−1^) also had no effect on protein synthesis, but when combined with the amino acids leucine, arginine and methionine there was almost a doubling of protein synthesis. The addition of carbohydrate (10.7 g kg^−1^) to stimulate insulin release had no additional effect. The combination involving the amino acids produced almost a doubling of the ratio of protein synthesis to protein degradation in gastrocnemius muscle over that of EPA alone. No treatment had a significant effect on tumour growth rate, but the inclusion of amino acids had a more significant effect on weight loss induced by the MAC16 tumour than that of EPA alone. The results suggest that combination therapy of cancer cachexia involving both inhibition of the enhanced protein degradation and stimulation of the reduced protein synthesis may be more effective than either treatment alone.

Atrophy of skeletal muscle in cancer cachexia results in weakness (asthenia) and death. Muscle mass is controlled by the rate of protein synthesis and the rate of protein degradation, which in the adult are normally in balance, so that muscle mass remains constant. In cachexia whole body protein turnover is increased (Carmichael *et al*, 1980), while in skeletal muscle protein synthesis is decreased (Lundholm *et al*, 1976), while protein degradation is elevated (Lundholm *et al*, 1982). Attempts to increase protein synthesis, for example, by elevation of substrate flux, in the absence of inhibitors of protein degradation have not increased lean body mass (Evans *et al*, 1985). Protein degradation in cancer cachexia has been attributed predominantly to an increased expression of the ubiquitin–proteasome proteolytic pathway (Lecker *et al*, 1999). One of the few agents capable of attenuating protein degradation in cachexia is the polyunsaturated fatty acid, eicosapentaenoic acid (EPA), which downregulates the increased gene expression of key regulatory components of the ubiquitin–proteasome pathway in skeletal muscle (Whitehouse *et al*, 2001). Although EPA attenuates the increased protein degradation in cancer cachexia, it has no effect on the depression of protein synthesis (Beck *et al*, 1991). Thus, EPA attenuates the development of weight loss in cachectic patients with pancreatic cancer, but weight gain is minimal (Wigmore *et al*, 2000). However, when patients were administered EPA together with a high-protein high-energy supplement weight gain was seen and this arose solely from an increase in lean body mass (Barber *et al*, 1999).

This suggests a mechanism to counter muscle atrophy in cancer cachexia by the inhibition of protein degradation, combined with a stimulus for protein synthesis. Muscle protein synthesis has been shown to be initiated by essential amino acids, particularly the branched chain amino-acid (BCAA) leucine (Anthony *et al*, 2000). Stimulation of protein synthesis requires an optimal plasma profile of amino acids and occurs through a mechanism that involves the stimulation of the eukaryotic initiation factor (eIF)-binding protein 1. Arginine is a conditionally essential amino acid, which has been shown to be deficient in tumour-bearing mice (Vissers *et al*, 2002). Arginine may improve the delivery of amino acids to muscle (Robertson *et al*, 1994), and thus may increase protein synthesis. Methionine is another essential amino acid, which may be rate limiting in protein synthesis. It has recently been shown (Tesseraud *et al*, 2003) that simultaneous addition of two amino acids, leucine and methionine, are critical in controlling protein translation. Methionine is not only a building block for proteins but is also the primary amino acid needed to initiate protein synthesis (Bradshaw *et al*, 1998). In addition, synthesis of acute phase proteins, which has been correlated with the loss of body mass in patients with lung and gastrointestinal cancers (McMillan *et al*, 1998), alters requirements for amino acids, since these proteins contain relatively high levels of sulphur-containing amino acids (Reeds *et al*, 1997). Lower plasma levels of leucine and methionine have been reported in the serum of weight-losing mice bearing the MAC16 tumour (Beck and Tisdale, 1989).

The purpose of the present study was to investigate the effect of protein (casein) and amino acids (leucine, arginine and methionine) together with EPA on protein synthesis and degradation in gastrocnemius muscle of mice bearing the MAC16 colon adenocarcinoma (Bibby *et al*, 1987). An additional group received carbohydrate, to stimulate insulin release, which should facilitate the uptake of amino acids into muscle and stimulate protein synthesis.

## MATERIALS AND METHODS

### Animals

Pure strain male NMRI mice were bred in our own colony and fed economy rodent breeder diet (Special Diet Services, Essex, UK) and water *ad libitum*. The diet contained 19.2% crude protein and 4.3% fat. Animals were implanted subcutaneously (s.c.) in the flank with fragments of the MAC16 tumour by means of a trochar, selecting from donor animals with established weight loss (Bibby *et al*, 1987). All animal experiments followed a strict protocol, approved by the British Home Office, and the ethical guidelines that were followed meet the standards required by the UKCCR guidelines (Workman *et al*, 1998). Weight loss was evident 10–12 days after transplantation and the animals were randomised into groups of 12 when the weight loss was about 5%. Six animals were used for protein synthesis and six for protein degradation. A nontumour-bearing group formed an additional control. Cachectic mice received olive oil as a control, EPA (1.0 g kg^−1^), EPA (1.0 g kg^−1^ and casein protein 5.35 g kg^−1^), EPA (1.0 g kg^−1^, casein 5.35 g kg^−1^, leucine 25.5 mg, arginine 12 mg, methionine 5 mg) and EPA (1.0 g kg^−1^, casein 5.35 g kg^−1^, amino acids as above and 10.7 g kg^−1^ carbohydrate). The supplements were administered twice daily in 50 *μ*l doses. All treatments were administered daily peritoneally (p.o.) by gavage for a 4-day period and protein synthesis and degradation were determined on the 4th day. The concentrations of protein, amino acids and carbohydrate were equivalent to those consumed by patients taking the recommended dosage of Resource® Support (Novartis Medical Nutrition). Tumour volumes were measured daily by means of calipers, and were recorded as a percentage of the starting tumour volume. Body weight was measured daily and recorded as the change in body weight from the start of the experiment. At the end of the experiment, the animals were humanely killed and the gastrocnemius muscles were rapidly removed for further analysis.

### Materials

L-[4^−3^H]Phenylalanine (specific activity 30 Ci mmol^−1^) was purchased from Amersham Life Science Products, Amersham UK. EPA as the synthetic triglyceride, Omegavie 90, contained 93.2% EPA as the fatty acid, and was purchased from Polaris, France. The carbohydrate was 10DE maltodextrin from Grain Processing Corporation, USA. The protein was a casein hydrolysate with the same amino-acid profile as casein and was purchased from New Zealand Milk Products.

### Protein synthesis and degradation in gastrocnemius muscle

The method for the determination of protein synthesis and degradation in gastrocnemius muscle has been described previously (Beck *et al*, 1991). Briefly, protein synthesis was measured by the incorporation of L-[4^−3^H]phenylalanine during a 2 h period in which isolated gastrocnemius muscles were incubated at 37°C in RPMI 1640 without phenol red and saturated with O_2_ : CO_2_ (19 : 1). After incubation, muscles were rinsed in nonradioactive medium, blotted and homogenised in 4 ml 2% perchloric acid. The rate of protein synthesis was calculated by dividing the amount of protein-bound radioactivity by the amount of acid-soluble radioactivity.

For protein degradation assays animals from the same group as used to measure protein synthesis were administered i.p. with 0.4 mM L-[4^−3^H]phenylalanine in phosphate-buffered saline (100 *μ*l) 24 h prior to the assay. Isolated gastrocnemius muscles were extensively washed with 0.9% NaCl and RPMI 1640 medium before measuring the release of radioactivity into RPMI 1640 over a 2 h period. The protein-bound radioactivity was determined by homogenising in 2% perchloric acid as above and the rate of protein degradation was calculated by dividing the amount of [^3^H]phenylalanine radioactivity released into the incubation medium during the 2 h incubation period by the specific activity of protein-bound [^3^H]phenylalanine.

### Statistical analysis

Results are expressed as mean±s.e.m. Differences were determined by one-way analysis of variance (ANOVA) followed by Tukey–Kramer multiple comparison test. *P*-values less than 0.05 were considered significant.

## RESULTS

**Figure 1:**
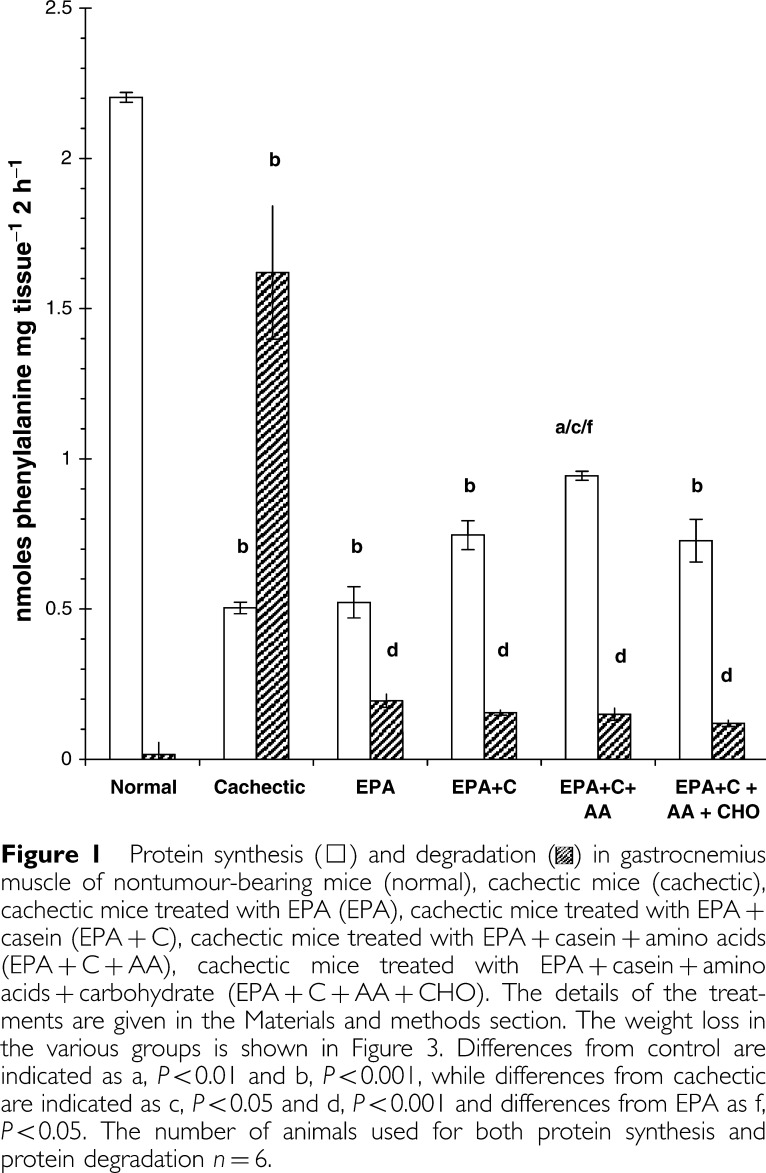
Protein synthesis (□) and degradation in gastrocnemius muscle of nontumour-bearing mice (normal), cachectic mice (cachectic), cachectic mice treated with EPA (EPA), cachectic mice treated with EPA+casein (EPA+C), cachectic mice treated with EPA+casein+amino acids (EPA+C+AA), cachectic mice treated with EPA+casein+amino acids+carbohydrate (EPA+C+AA+CHO). The details of the treatments are given in the Materials and methods section. The weight loss in the various groups is shown in Figure 3. Differences from control are indicated as a, *P*<0.01 and b, *P*<0.001, while differences from cachectic are indicated as c, *P*<0.05 and d, *P*<0.001 and differences from EPA as f, *P*<0.05. The number of animals used for both protein synthesis and protein degradation *n*=6.

The effects of the various treatments on protein synthesis and protein degradation in gastrocnemius muscle of cachectic mice bearing the MAC16 tumour is shown in Figure 1

**Figure 2:**
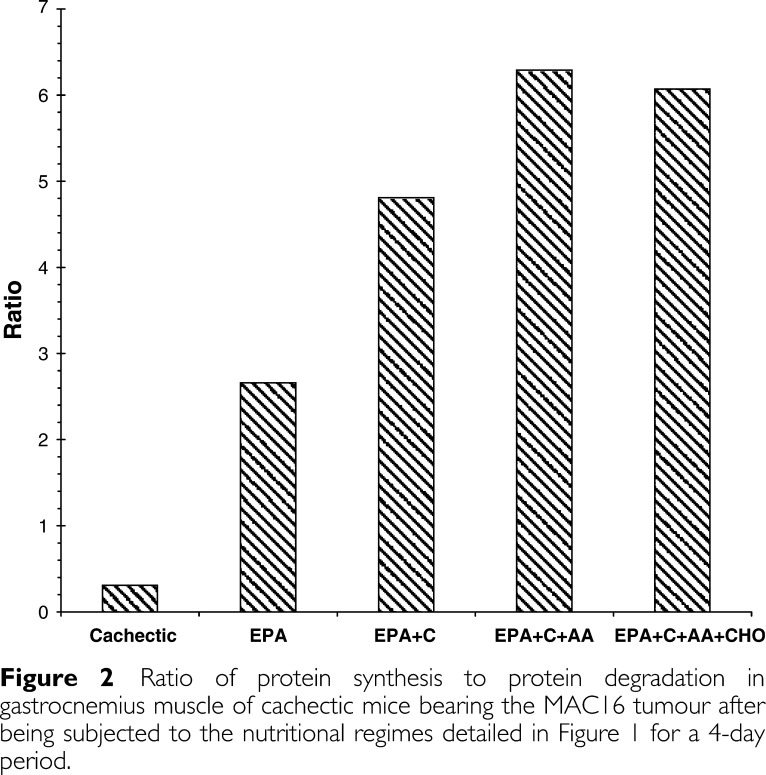
Ratio of protein synthesis to protein degradation in gastrocnemius muscle of cachectic mice bearing the MAC16 tumour after being subjected to the nutritional regimes detailed in Figure 1 for a 4-day period.

. The concentration of EPA used was suboptimal in order to discern synergistic interactions. Control animals showed high levels of protein synthesis and little protein degradation, which was expected, since they were growing steadily. There was a significant depression in protein synthesis and a large increase in protein degradation in muscles of cachectic mice bearing the MAC16 tumour, as reported previously (Beck *et al*, 1991). EPA treatment significantly reduced protein degradation, but had no effect on protein synthesis. There was no significant effect of any of the other treatments on protein degradation compared with that of EPA alone. There was a small, but nonsignificant elevation in protein synthesis in animals receiving casein in addition to EPA. However, when casein was administered together with leucine, arginine and methionine, there was a significant increase in protein synthesis above that seen in cachectic mice or cachectic mice administered EPA. Interestingly, this effect was not seen in the presence of carbohydrate. The ratio of protein synthesis to degradation was improved by all supplement treatments over that of EPA alone (Figure 2

), although it never reached the values observed in nontumour-bearing mice.

**Figure 3:**
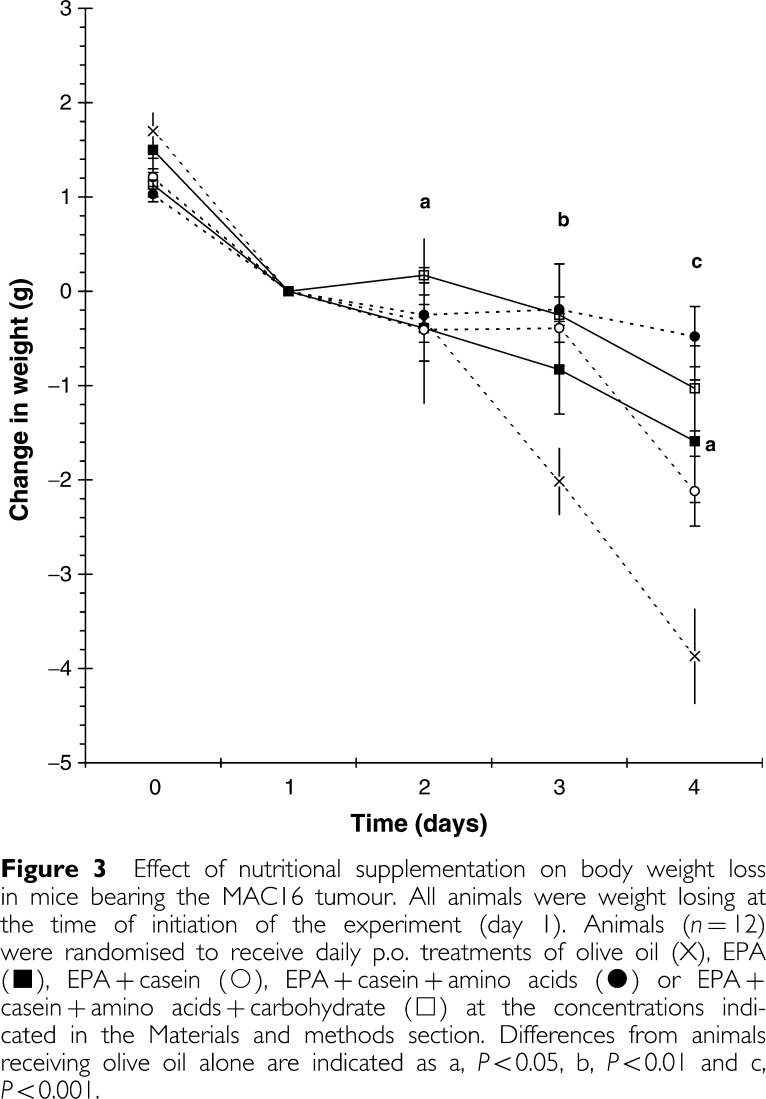
Effect of nutritional supplementation on body weight loss in mice bearing the MAC16 tumour. All animals were weight losing at the time of initiation of the experiment (day 1). Animals (*n*=12) were randomised to receive daily p.o. treatments of olive oil (X), EPA (▪), EPA+casein (○), EPA+casein+amino acids (•) or EPA+casein+amino acids+carbohydrate (□) at the concentrations indicated in the Materials and methods section. Differences from animals receiving olive oil alone are indicated as a, *P*<0.05, b, *P*<0.01 and c, *P*<0.001.

The effect of the various treatments on body weight loss is shown in Figure 3

**Figure 4:**
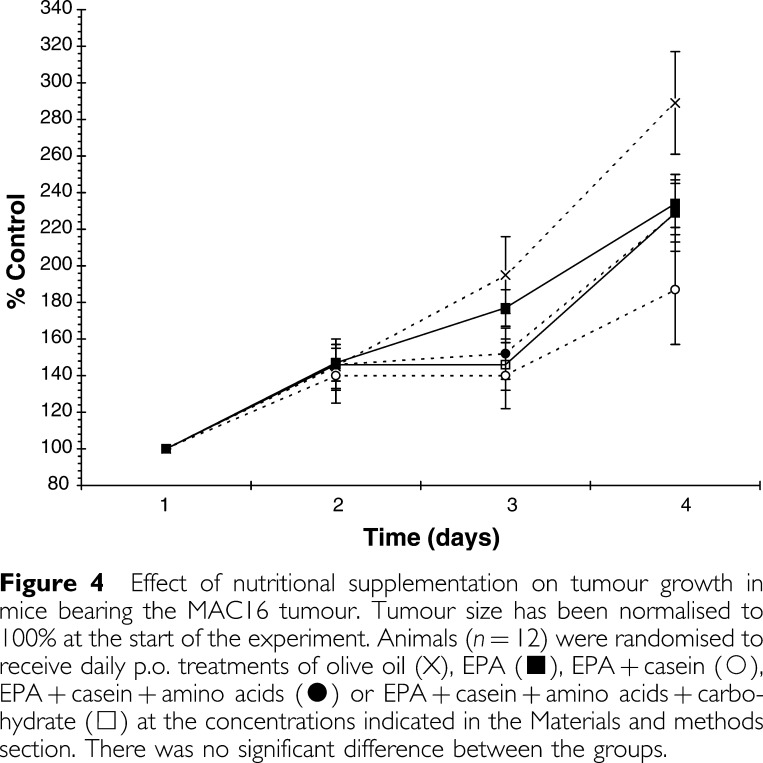
Effect of nutritional supplementation on tumour growth in mice bearing the MAC16 tumour. Tumour size has been normalised to 100% at the start of the experiment. Animals (*n*=12) were randomised to receive daily p.o. treatments of olive oil (X), EPA (▪), EPA+casein (○), EPA+casein+amino acids (•) or EPA+casein+amino acids+carbohydrate (□) at the concentrations indicated in the Materials and methods section. There was no significant difference between the groups.

. Both EPA and EPA+casein reduced total body weight loss, but this did not reach significance except at the 4-day time point. However, the addition of both amino acids and amino acids+carbohydrate to the EPA+casein regime caused a significant reduction in weight loss compared with nontreated controls. None of the treatments had a significant effect on tumour volume (Figure 4

).

## DISCUSSION

The mechanism for the increased protein degradation in skeletal muscle in cancer cachexia has been suggested as an increased expression of the ubiquitin–proteasome proteolytic pathway (Williams *et al*, 1999). Various cytokines, such as tumour necrosis factor-*α* or interferon gamma (Llovera *et al*, 1998), have been shown to increase transcripts of ubiquitin in skeletal muscle, but the importance of these cytokines as mediators of the weight loss in human cancer cachexia is controversial (Maltoni *et al*, 1997). In cancer cachexia, a sulphated glycoprotein, proteolysis-inducing factor (PIF) produced by cachexia inducing tumours (Todorov *et al*, 1996) may be more important in skeletal muscle atrophy. Proteolysis-inducing factor stimulates loss of the myofibrillar protein myosin directly by increasing the expression of proteasome subunits and the ubiquitin-conjugating enzyme (E2_14k_) (Gomes-Marcondes *et al*, 2002). The process is initiated by the activation of PIF of an intracellular signalling cascade in muscle involving the release of arachidonic acid from membrane phospholipids and metabolism to 15-hydroxyeicosatetraenoic acid (15-HETE) (Smith *et al*, 1999), and involving the activation of the transcription factor nuclear factor-*κ*B (NF-*κ*B) (Whitehouse and Tisdale, 2003). Eicosapentaenoic acid attenuates this process, both by inhibiting the formation of 15-HETE (Smith *et al*, 1999) and by stabilising the I*κ*B/NF-*κ*B cytosolic complex and nuclear accumulation of NF-*κ*B (Whitehouse and Tisdale, 2003), preventing the increase in proteasome expression and degradation of myofibrillar proteins. Proteasome-mediated proteolysis is independent of the amount of protein consumed, so that simple nutritional supplementation would not be expected to prevent muscle catabolism as observed in mice bearing the MAC16 tumour. However, leucine has been shown to inhibit the expression of genes of the proteasome pathway in muscle of cachectic rats, but not in those of rats after starvation (Busquets *et al*, 2002). Branched chain amino acids also directly inhibit the proteasome ‘chymotrypsin-like’ enzyme activity, the predominant proteolytic activity of the *β*-subunits of the proteasome in skeletal muscle, but not in the liver (Hamel *et al*, 2003). Despite this in the present study, there was no evidence for attenuation of protein degradation by leucine/amino-acid mixture below that induced by EPA alone in gastrocnemius muscle of cachectic mice bearing the MAC16 tumour.

In addition to the effect on protein degradation PIF also inhibits protein synthesis in muscle, but the mechanism by which this occurs is not known, although it is attenuated by insulin at physiological concentrations and below, but not by EPA (Smith *et al*, 1999). The action of insulin suggests that PIF may inhibit translation, for example, by dephosphorylation of translation factors or the S6 ribosomal protein. Branched chain amino-acid, and leucine in particular, enhance muscle protein synthesis through the activation of the mRNA-binding step in translation initiation in skeletal muscle, but not in the liver (Yoshizawa, 2004). The action of leucine is mediated by the activation of the ribosomal protein S6 kinase and by phosphorylation of initiation factors, suggesting that it may, like insulin, overcome the PIF-induced inhibition of protein synthesis. This study shows that while casein alone did not significantly stimulate protein synthesis in gastrocnemius muscle of cachectic mice, there was a significant increase in protein synthesis with the addition of the leucine/arginine/methionine mix. This was not further enhanced by the carbohydrate mix, although leucine and arginine have been previously shown to stimulate insulin secretion (Malaisse, 1984), and insulin plays a permissive role in leucine-induced protein synthesis (Yoshizawa, 2004). Thus treatment of cancer patients with a combination of EPA, protein and amino acids, particularly leucine, would be expected to attenuate protein degradation and stimulate protein synthesis in skeletal muscle, which should lead to improvements both in the quality of life and survival, and this combination is currently under clinical evaluation.

## References

[bib1] Anthony JC, Anthony TG, Kimball SR, Vary TC, Jefferson IS (2000) Orally administered leucine stimulates protein synthesis in skeletal muscle of postabsorptive rats in association with increased eIF4F formation. J Nutr 130: 139–145 10720160 10.1093/jn/130.2.139

[bib2] Barber MD, Ross JA, Voss AC, Tisdale MJ, Fearon KCH (1999) The effect of an oral nutritional supplement enriched with fish oil on weight-loss in patients with pancreatic cancer. Br J Cancer 81: 80–86 10487616 10.1038/sj.bjc.6690654 PMC2374349

[bib3] Beck SA, Smith KL, Tisdale MJ (1991) Anticachectic and antitumor effect of eicosapentaenoic acid and its effect on protein turnover. Cancer Res 51: 6089–6093 1657378

[bib4] Beck SA, Tisdale MJ (1989) Nitrogen excretion in cancer cachexia and its modification by a high fat diet in mice. Cancer Res 49: 3800–3804 2736521

[bib5] Bibby MC, Double JA, Ali SA, Fearon KCH, Brennan RA, Tisdale MJ (1987) Characterization of a transplantable adenocarcinoma of the mouse colon producing cachexia in recipient animals. J Natl Cancer Inst 78: 539–546 3546909

[bib6] Bradshaw RA, Brickley WN, Walker KW (1998) N-terminal processing: the methionine aminopeptidase and *N*-alpha-acetyl transferase families. Trends Biochem Sci 23: 263 9697417 10.1016/s0968-0004(98)01227-4

[bib7] Busquets S, Alvarez B, Lopez-Soriano FJ, Argiles JM (2002) Branched-chain amino acids: a role in skeletal muscle proteolysis in catabolic states? J Cell Physiol 19: 283–289 10.1002/jcp.10097 12012323

[bib8] Carmichael MJ, Clague MB, Keir MJ, Johston ID (1980) Whole body protein turnover, synthesis and breakdown in patients with colorectal carcinoma. Br J Surg 67: 736–739 7427029 10.1002/bjs.1800671015

[bib9] Evans WK, Makuch R, Clamon GH, Feld K, Weiner RS, Moran E, Blum R, Shepherd FA, Jeejeebhoy KW, De Wys WD (1985) Limited impact of total parenteral nutrition on nutritional status during treatment for small cell lung cancer. Cancer Res 45: 3347–3353 2988769

[bib10] Gomes-Marcondes MCC, Smith HJ, Cooper JC, Tisdale MJ (2002) Development of an *in-vitro* model system to investigate the mechanism of muscle protein catabolism induced by proteolysis-inducing factor. Br J Cancer 86: 1628–1633 12085214 10.1038/sj.bjc.6600236 PMC2746596

[bib11] Hamel FG, Upward JL, Siford GL, Duckworth WC (2003) Inhibition of proteasome activity by selected amino acids. Metabolism 52: 810–814 12870152 10.1016/s0026-0495(03)00094-5

[bib12] Lecker SH, Solomon V, Mitch WE, Goldberg AL (1999) Muscle protein breakdown and the critical role of the ubiquitin–proteasome pathway in normal and disease states. J Nutr 129: 227S–237S 9915905 10.1093/jn/129.1.227S

[bib13] Llovera M, Carbo N, Lopez-Soriano J, Garcia-Martinez C, Busquets S, Alvarez B, Agell N, Costelli P, Lopez-Soriano FJ, Celada A, Argiles JM (1998) Different cytokines modulate ubiquitin gene expression in rat skeletal muscle. Cancer Lett 133: 83–87 9929164 10.1016/s0304-3835(98)00216-x

[bib14] Lundholm K, Bylund AC, Holm J, Schersten T (1976) Skeletal muscle metabolism in patients with malignant tumour. Eur J Cancer 12: 465–473 182443 10.1016/0014-2964(76)90036-0

[bib15] Lundholm K, Bennegard K, Eden E, Rennie MJ (1982) Efflux of 3-methylhistidine from the leg of cancer patients who experience weight loss. Cancer Res 42: 4809–4818 7127315

[bib16] Malaisse WJ (1984) Branched-chain amino and keto acids as regulators of insulin and glucagon release. In Branched Chain Amino and Keto Acids in Health and Disease, Adibi SA, Fekl W, Langenbeck U, Schauder P (eds) pp 119–133, Switzerland: Karger, Basel

[bib17] Maltoni M, Fabbri L, Nanni O, Scarpi E, Pezzi L, Flamini E, Riccobon A, Derni S, Pallotti G, Amadori D (1997) Serum levels of tumour necrosis factor alpha and other cytokines do not correlate with weight loss and anorexia in cancer patients. Support Care Cancer 5: 130–135 9069613 10.1007/BF01262570

[bib18] McMillan DC, Scott HR, Watson WS, Preston T, Milroy R, McArdle CS (1998) Longitudinal study of body cell mass depletion and the inflammatory response in cancer patients. Nutr Cancer 31: 101–105 9770720 10.1080/01635589809514687

[bib19] Reeds PJ, Fjeld CR, Jahoor F (1997) Do the differences between the amino acid compositions of acute phase and muscle proteins have a bearing on nitrogen loss in traumatic states? J Nutr 124: 906–910 10.1093/jn/124.6.906 7515956

[bib20] Robertson FM, Offner PJ, Ciceri DP, Becker WK, Pruitt BA (1994) Detrimental hemodynamic effect of nitric oxide synthase inhibition in septic shock. Arch Surg 129: 149–155 7508219 10.1001/archsurg.1994.01420260045005

[bib21] Smith HJ, Lorite MJ, Tisdale MJ (1999) Effect of a cancer cachectic factor on protein synthesis/degradation in murine C_2_C_12_ myoblasts: modulation by eicosapentaenoic acid. Cancer Res 59: 5507–5513 10554027

[bib22] Tesseraud S, Bigot K, Toovis M (2003) Amino acid availability regulates S6K1 and protein synthesis in avian insulin-insensitive QMT myoblasts. FEBS Lett 540: 176–181 12681504 10.1016/s0014-5793(03)00260-6

[bib23] Todorov P, Cariuk P, McDevitt T, Coles B, Fearon K, Tisdale M (1996) Characterization of a cancer cachectic factor. Nature 379: 739–742 8602222 10.1038/379739a0

[bib24] Vissers YLJ, von Meyenfeldt MF, Deutz MEP (2002) Arginine production in intestine, liver, muscle and kidney is reduced in tumour-bearing mice undergoing surgery. Eur J Gastroenterol Hepatol 14: A59–A64

[bib25] Whitehouse AS, Smith HJ, Drake JL, Tisdale MJ (2001) Mechanism of attenuation of skeletal muscle protein catabolism in cancer cachexia by eicosapentaenoic acid. Cancer Res 61: 3604–3609 11325828

[bib26] Whitehouse AS, Tisdale MJ (2003) Increased expression of the ubiquitin–proteasome pathway in murine myotubes by proteolysis-inducing factor (PIF) is associated with activation of the transcription factor NF-*κ*B. Br J Cancer 89: 1116–1122 12966435 10.1038/sj.bjc.6601132 PMC2376944

[bib27] Wigmore SJ, Barber MD, Ross JA, Tisdale MJ, Fearon KCH (2000) Effect of oral eicosapentaenoic acid on weight loss in patients with pancreatic cancer. Nutr Cancer 36: 177–184 10890028 10.1207/S15327914NC3602_6

[bib28] Williams A, Sun X, Fischer JE, Hasselgren P-O (1999) The expression of genes in the ubiquitin–proteasome proteolytic pathway is increased in skeletal muscle from patients with cancer. Surgery 126: 744–750 10520924

[bib29] Workman P, Twentyman P, Balkwill F, Balmain A, Chaplin D, Double J, Embelton J, Newell D, Raymond R, Stables J, Stephens T, Wallace J (1998) United Kingdom Co-ordinating Committee on Cancer Research (UKCCR). Guidelines for the welfare of animals in experimental neoplasia (second edition). Br J Cancer 77: 1–10 10.1038/bjc.1998.1 PMC2151254 9459138

[bib30] Yoshizawa F (2004) Regulation of protein synthesis by branched-chain amino acids *in vivo*. Biochem Biophys Res Commun 313: 417–422 14684178 10.1016/j.bbrc.2003.07.013

